# Civic Duty: A Booster for Resilience?

**DOI:** 10.3389/ijph.2021.1604064

**Published:** 2021-06-30

**Authors:** Pauline Yongeun Grimm, Dell D. Saulnier

**Affiliations:** ^1^ Swiss Centre for International Health, Swiss Tropical and Public Health Institute (Swiss TPH), Basel, Switzerland; ^2^ University of Basel, Basel, Switzerland; ^3^ Department of Global Public Health, Karolinska Institutet, Stockholm, Sweden

**Keywords:** COVID-19, resilience, civic duty, crisis management, collaborative governance

**The IJPH series “Young Researcher Editorial” is a training project of the Swiss School of Public Health.**

Though the COVID-19 pandemic has taken centre stage in our lives, climate change, conflict, and economic recession still threaten our systems and societies. The capacity of systems and societies to absorb, adapt, and transform when exposed to a shock and still retain their core functions is called resilience. Building resilience is one way of managing such crises. One important driver of resilience may be social capital [1], which plays an important role during times of crisis and calm [2]. A component of social capital is civic capital. Civic capital comprises values that support cooperation for the common good and civic engagement. We argue that civic capital is a key ingredient in resilience and that communities, health systems, and governments require civic capital to effectively manage and recover from current and future crises.

Communities with more trust, civic engagement, and tighter networks, can recover better than more fragmented counterparts after a crisis. Civic capital increases trust among community members, leads to more sharing of critical information and cooperative partnerships, which seems to be a foundation for resilience [3]. The role of civic capital in the recovery process is evident in the aftermath of three major disasters: the Kobe Earthquake, Hurricane Katrina, and the Indian Ocean Tsunami. Proxy indicators to measure the strength of civic capital were levels of trust in fellow citizens and government institutions, time and energy spent on civic duties, and the ability of citizens to mobilize cooperatively. Despite drastic differences in income levels, people in Kobe and India had more civic capital than people in New Orleans, mirroring the efficiency and sustainability of their recoveries [3].

The social contract that promotes civic capital may be shaped by the definition of a “successful” response to a crisis like the COVID-19 pandemic. Many governments responded to COVID-19 by prioritizing goals like suppressing transmission or “flattening the curve” and used metrics like number of cases, reproductive rate, and occupancy rate of intensive care beds as indicators of progress and success. In some cases, stringent interventions like lockdowns and cancellation of routine healthcare appointments took a heavy toll on the health of non-COVID patients. For example, it marginalized cancer care and reduced support for other chronic conditions [4]. Countries that took a top-down authoritarian approach may have successfully suppressed transmission over the short term at the expense of community ownership, eroding the vital precondition of trust that legitimizes resilient systems [5].

Another example of civic capital is that of South Korea, one of the few countries that neither introduced a formal lockdown nor closed its borders. Instead, South Korea focused on meticulous contact tracing and strict, consistently applied quarantine rules. In combination with active civic engagement, these strategies effectively contained further spread of the virus [6]. During the first few months of the pandemic from January to May 2020, the trust index for the government of South Korea increased by 16% and its citizens expected the government to take the lead in the pandemic response [7]. This coincides with high levels of individual adherence to public prevention protocols. Choi attributes South Korea’s success to the synergy of collaborative governance: civil society and the public strongly determine the effectiveness of this system [8].

While Sweden followed the same strategy as many countries to “flatten the curve,” the Public Health Agency mainly implemented voluntary measures, in line with Swedish law, which prioritizes voluntary public health measures and limits the government’s power to set sweeping national restrictions [9]. In Sweden, the Public Health Agency’s recommendations are assumed as a citizen’s civic duty. Social trust is a long-standing value in Swedish culture [10] and along with contextual factors like comprehensive sick leave policies, this trust increased cooperation during the pandemic without more restrictive measures. Relying on civic duty alone, however, is insufficient: Sweden struggled to coordinate COVID-19 health response across administrative levels, which contributed to the failure of the government’s strategy to protect the elderly [11].

A society with stronger civic capital has the advantage of trusting in the government in its institutions, and the ability to rely on shared values and norms that support collective governance in times of crises and calm. This legitimacy fosters change and lets societies and systems learn; it is the foundation of resilience. The drivers of civic capital in a society are complex. A society is motivated by its history, constitution, culture, religion, and social norms. Scholars have shown that civic capital can grow if a society empowers self-initiated local groups and involves them in decision-making [12]. As an example, tax incentives have increased participation in community-based groups and changes in village layout have improved network and trust within communities [3]. Governments may find that identifying and customizing contextually relevant policy levers that build civic capital are worthwhile endeavours that increase their country’s resilience in the next crisis.
